# Is the use of antibiotic stewardship measures in the context of specialized outpatient palliative care sensible and feasible? An interview-based study

**DOI:** 10.1186/s12904-024-01609-x

**Published:** 2024-12-07

**Authors:** Ulrich Kaiser, Florian Kaiser, Jörg Schmidt, Ursula Vehling-Kaiser, Florian Hitzenbichler

**Affiliations:** 1 Clinic and Polyclinic for Internal Medicine III, University Hospital Regensburg, Franz-Josef-Strauß-Allee 11, 93053 Regensburg, Germany; 2 MVZ Dr. Vehling-Kaiser GmbH, Landshut, Germany; 3 Institute for Market Research in Healthcare, Munich, Germany; 4 Department of Infection Prevention and Infectious Diseases, University Hospital Regensburg, Regensburg, Germany

**Keywords:** SAPV, ABS, Infection, Palliative patient, Antibiotic therapy

## Abstract

**Background:**

Specialized outpatient palliative care (SAPV) is a component of palliative care in Germany, which assists approximately 10% of palliative patients. The majority of these patients have a malignant disease and are at increased risk of complications or severe infection. Antibiotic stewardship (ABS) measures are implemented to optimize antibiotic administration; however, there is little data available in this area, particularly for SAPV. Therefore, we examined the extent to which ABS measures can be meaningfully used or implemented in SAPV.

**Methods:**

After establishing a corresponding interview guide, 15 experts from specialized areas were interviewed on this subject by the Institute for Market Research in Healthcare Munich (IMIG) through audio-registered individual interviews. The interviews were analyzed using the qualitative content analysis method according to Mayring.

**Results:**

All 15 experts participated. The primary benefits cited were greater safety in the prescription and decision-making process for antibiotics in the areas of SAPV and improved quality of life. The implementation of continuous ABS measures for SAPV was considered difficult in some cases and linked to certain prerequisites, such as supportive advice from existing systems. The possibility of further training for SAPV members in the area of ABS was considered particularly advantageous.

**Conclusions:**

The implementation of ABS measures in SAPV is feasible in principle; however, it is difficult to implement under the current conditions. Close cooperation with an existing external ABS expert/team will be helpful. This will provide more security for a small, but relevant proportion of SAPV patients, and for the SAPV team treating them.

**Supplementary Information:**

The online version contains supplementary material available at 10.1186/s12904-024-01609-x.

## Background

With over 400 palliative care teams, specialized outpatient palliative care (SAPV) is an important element of palliative care in Germany [1]. The goal of SAPV is to maintain the quality of life and self-determination of palliative patients so they can remain in their home environment until the end of their lives [2]. Because of the complexity of their illness (e.g., pain, severe pulmonary, cardiac, gastroenterological, or neurological problems, ulcerative wounds), approximately 10% of palliative care outpatients require co-care by SAPV [3]. Approximately 70%–80% of these patients receiving SAPV care suffer from a malignant disease [2]. This group is particularly at risk for complications or severe courses of infections because of the existing underlying disease and/or previous antiproliferative drug regimens [4]. The most common infections are pulmonary and urinary tract infections with symptoms, such as a nagging cough, shortness of breath, and dysuria [4]. Moreover, chronic infections and ulcerating wounds not only lead to local problems, but often result in the isolation of the affected patient [4]. Despite treatment recommendations [5], the use of antibiotics in a palliative setting is controversial discussed [6–8]. Factors influencing the treatment decision include the course of the disease, life expectancy, current symptoms, stress, and the patient’s wishes [7, 8]. In addition, the wishes, expectations, or demands of relatives and different views/procedures of the co-treatment professional colleagues regarding the use of antibiotics must also be considered [7]. The appropriate, prudent, and controlled use of antibiotics is a core objective of antibiotic stewardship (ABS) as indicated in the current guidelines. This includes measures and strategies ranging from the determination of indications to the setting of dosage, duration of therapy and changes in therapy. The objectives include improving the quality of therapy and reducing the development of resistance [9]. Experts specially trained in the use of antibiotics are available to answer complex questions (e.g., as part of an interdisciplinary ABS visit); however, these often refer to an inpatient setting – also in the field of palliative care [10]. Therefore, the question arises as to whether SAPV, which also involves far-reaching decisions regarding the use of antibiotics in complex care situations, would benefit from ABS system. There is little data available in this regard, particularly in the area of SAPV. In this study, we examined the extent to which ABS measures can be meaningfully used or implemented in SAPV.

## Methods

After consultation with the Ethics Committee of the University of Regensburg, no approval was required for the present study.

In October 2023, a team of experts (palliative care physicians, oncologists, SAPV staff, and ABS experts) developed a semistructured interview guide based on the literature for the development of semi-structured interviews (Gesis-Guidelines) [11–13], existing literature on antibiotics in palliative care [4, 8, 14–16] and personal experience, which consisted of 26 open questions. The interview guide is attached as a supplementary file. Before the interview guide was used, pre-testing was carried out in line with current recommendations for validating functionality [17]. Our goal was to clarify the expert attitude toward ABS measures and determine the possibility/usefulness of implementing ABS measures in SAPV. Another goal was to get knowledge about antibiotic use in palliative care/SAPV.

Subsequently, 15 experts (f: n = 5, m: n = 10, Table 1; sufficient data saturation was achieved after 15 interviews) took part in the interviews. Of these experts, eight were active in SAPV. In order to cover as many different experiences and perspectives as possible and thereby to generate statements on the feasibility of ABS in SAPV that are as differentiated as possible, the experts were selected according to the following criteria: Expertise in ABS, palliative care and/or SAPV, ethical-palliative care aspects, haematology/oncology, general practitioner care of palliative patients as well as organization and quality assurance in SAPV. The participants worked both in the university inpatient sector (infectiology/microbiology, hematology/oncology, palliative medicine) and in the outpatient sector (SAPV, hematology/oncology, general medicine). In particular, experts who are primarily involved in the care of palliative patients (in addition to SAPV; especially general practitioner and hematologists/oncologists) were also included in the survey. After providing information about the study as well as written consent, the interviews were conducted in German by the Institute for Market Research in Healthcare Munich (IMIG) from October 2023 to January 2024. IMIG is a specialized research institution and has many years of experience in conducting qualitative interviews. This ensured a high level of methodological quality. None of the authors took part in the study as an interviewed participant/expert in order to avoid any preconceived opinions.

**Table 1:** Specialties of the interview participants

Specialization	Number (n)
Specialist in oncology and hematology	2
Specialist in oncology/hematology/palliative medicine/ethics consultant	1
Specialist in oncology/hematology/palliative medicine	2
Specialist in family medicine/palliative medicine	1
Specialist in urology/palliative medicine	1
Psychologist with specialization in psycho-oncology	1
Specialist in microbiology, virology, and infection epidemiology	1
SAPV nursing care	1
Hospice palliative nurse	1
Specialist in internal medicine and infection, ABS expert	1
Quality management	1
Palliative care nurse	2

Before the interviews, each participant was informed of the content and objectives of ABS measures and SAPV to guarantee a uniform level of information. Participants had the option to terminate their participation in the study at any time. Individual interviews were conducted via Microsoft Zoom with a target length of 60 min, although there was no fixed time limit. The interviewees were provided sufficient time to answer the questions. The interviews were audiographically recorded. All participants were fully informed of the study and written consent was obtained before each interview. The documentation and evaluation were anonymous. To ensure high methodological quality and to guarantee an adequate survey quality for the interview participants, IMIG was commissioned as an independent institution to conduct the interviews, which took place from October 2023 to January 2024.

Semistructured qualitative interviews are conducted individually by an interviewer with one interviewed participant. They are based on an interview guide and on an open discussions. This ensures an atmosphere in which the interview participants can express all relevant opinions and thoughts on the topic. This leads to meaningful results that are typical for the respective target group [18–21]. In addition, the interviewer can ask questions directly if necessary and use additional questions to further obtain relevant information or expand on it accordingly. This method of data collection was chosen for our study in order to generate the most comprehensive, multi-layered and differentiated range of results.

The interviews were recorded and transcribed by IMIG for further processing. The responses were analyzed using the qualitative content analysis method according to Mayring [21]. This is a multi-stage, structured, reproducible analysis procedure, in which the steps include the categorization, coding, re-evaluation, and analysis of the interviews, thus systematically and transparently abstracting the qualitative data. After an initial review of the material, the main topics of the interview guide were adopted as the a priori main category system. During the analysis process of the interview data, subcategories were successively added to the main categories by summarizing and identifying the most important topics and the (sub)category system was successively expanded to include new codes and concepts. The categories were constantly re-evaluated and adjusted as new findings emerged. To ensure interpersonal validity, three researchers conducted and discussed the analytic process. Finally, the results were summarized and evaluated. In addition, verbatim statements (quotes) from the interviewees were included in the analysis in an anonymized form, which highlighted how the interviewees thought and expressed themselves.

## Results

All of the experts consulted took part in the study. The interviews lasted 45–60 min. The results of the individual categories are described below. Quotes from SAPV employees are marked with *SAPV* and all others are marked *non-SAPV*.

## Use of antibiotic therapy in palliative care in general

In contrast to the curative setting, all respondents viewed the use of antibiotics in the final phase of life as critical because “you take action in the late palliative phase because otherwise you can't cope, you say you feel better when you do something that has no therapeutic effect” (quote *SAPV, Participant 13*). In the early phase of palliative care, 12 participants weighed in on the pros and cons of using antibiotics. The focus here is symptom relief; thus, the goal is to improve the palliative patient’s quality of life. Three participants were unable to make a clear decision on this question.

Quote (*non-SAPV, Participant 2*)*:* “The advantage is the elimination of symptoms and a better quality of life.”

However, the issue of prolonging life is also addressed in the context of antibiotic use.

Quote (*SAPV, Participant 5*)*:* “The advantages, of course, are that you support the patient, it’s about symptom control and also about prolonging life, I think that’s an advantage.”

The form of administration “i.e. when, for example, swallowing tablets or juices becomes more difficult” (quote *SAPV, Participant 12*), and the more difficult diagnostics in the palliative setting were also noted by the interview participants.

The disadvantages of antibiotic use include side effects, interactions with other medications, and “organ stress and additional tablets” (quote *SAPV, Participant 1*).

### Use of antibiotics in SAPV

The use of antibiotics in SAPV (apart from the final phase) was viewed positively by nine respondents, ambivalently by four, and critically by two. The respondents’ statements ranged from “It is very important…” (quote *SAPV, Participant 9*) to “It really is a niche drug that is used in individual cases” (quote *SAPV, Participant 5*).

All respondents were in favor of a primary benefit/risk assessment when using antibiotics. In addition to the patient’s condition and life expectancy, other aspects, such as side effects, interactions, form of administration, and the patient’s wishes should also be considered.

Quote (*SAPV, Participant 9*)*: "We in SAPV also have patients who are still in a good general condition and have a good quality of life. And at this stage, I wouldn't deny anyone an antibiotic. …we have to respect what the patient wants. And if they want symptom relief for a long time … then we don't have the right to change that."*

In addition to rapid symptom control and an improved quality of life, an improvement in prognosis was also cited as a benefit to antibiotic administration.

Quote (*non-SAPV, Participant 2*)*:* “The advantages are the reduction of symptoms, improvement in well-being, perhaps also an improvement in the prognosis for some infections.”

Regarding the disadvantages of administering antibiotics in SAPV, in addition to side effects/interactions and the intake of additional medications, there was also an additional time component: “… a lot of educational work is necessary …” (quote *SAPV, Participant 12*).

In response to the question of how this should be handled if there are differing views on the use of antibiotics between the SAPV team, the patient, their relatives, or the patient’s family doctors/specialists providing parallel treatment, it is recommended that open discussions be held with all parties involved and that coordination with the treating physicians be sought.

Only 6 of the 15 people interviewed would admit a patient from SAPV as an inpatient because of an infection. Nine interviewees were ambivalent about the issue of hospitalization of palliative patients and would make the decision based on the patient’s condition.

### Attitude and evaluation of ABS

Thirteen of the 15 respondents considered the topic ABS as offered and implemented in clinics clearly positive and very useful.

Quote (*non-SAPV, Participant 7*)*:* “So antibiotic stewardship, I think these programs are an important way of ensuring rational use of antibiotics ….”

There were several reasons for this, such as the higher quality of antibiotic therapy, the reduced incidence of drug resistance, the professional training of the team in the clinic, and the possibility of using antibiotics rationally and appropriately in the future.

Two interviewees had an ambivalent view toward this topic as they considered the scope to be too large and costly, although they saw advantages to the training courses.

Quote (*SAPV, Participant 5*)*:* “I don't know whether this huge amount of work is relevant in relation to the situation. Somehow these measures are too extensive for me. I think this training is quite good, that you differentiate more ….”

Nine interviewees already had experience with ABS in their professional lives because they were working in a clinic where the topic was present or because they had contact with it through previous professional contacts in clinics.

The primary benefits for the participants in the ABS training courses and through the ABS measures taught were the high level of competence in handling antibiotics and the associated transfer of this knowledge, followed by increased sensitization and awareness of the topic of antibiotic therapy in the clinic.

Quote (*non-SAPV, Participant 10*): “So antibiotic stewardship, I think these programs are an important way of ensuring rational use of antibiotics. …. we have already seen in pilot projects that training the nursing staff is very important here. The contact between nursing staff and patients is much greater than between physician and patients. This means that nursing staff will in many cases be diagnostic triggers, which in many cases also trigger the administration of antibiotics.”

The primary benefits for patients in hospitals where ABS training courses and ABS measures are carried out according to all 15 interviewees included more targeted, better antibiotic therapy, faster rate of healing success, and reduced length of inpatient stay. The reduced occurrence of side effects from antibiotics was also cited as a benefit.

Quote (*non-SAPV*, *Participant 7)*: “It is essential… in everyday patient care. We are limited when it comes to selecting new substances for the treatment of infectious diseases and every antibiotic therapy leads to a shift in the microbiome and the development of resistance, and everything we do to avoid this makes sense for the targeted use of antibiotics.”

Some respondents cited the increased diagnostic workload, such as blood collection, imaging procedures, or microbiological tests, as disadvantages for patients as a result of the ABS training courses and ABS measures.

### ABS in SAPV

#### Pros and cons of ABS system/ABS measures in SAPV

Five participants were positive about the introduction of an ABS system/ABS measure in SAPV, whereas 10 participants viewed it critically.

The general benefit for palliative patients with a higher risk of infection, an improvement in the quality of treatment, particularly for “problem patients,” and a better knowledge of the respective pathogen spectrum were considered positive.

Quote (*SAPV, Participant 11*): “I think that's a very good idea in itself, because we also need similar structures. And then there should also be a standardization of therapy. I think that's a very good idea in itself.”

Quote (*non-SAPV, Participant 2*)*:* “This is not my top priority, but a good knowledge of the patient's pathogen spectrum is relevant, especially in terms of palliative care.”

The possibility of lowering costs through targeted antibiotic therapy was also addressed.

Quote (*SAPV, Participant 11*)*:* “A very elementary part is that every medical prescription entails costs and so something like this would also save costs. If you choose a more expensive antibiotic because it's compatible with the testing and I don't need a second one and an additional test, it's also better for the quality of life. But as I said, it's definitely also a cost saving within the budget.”

The following were frequently cited as important points with regard to the introduction and regular implementation of ABS measures in SAPV:

- 1) Too much time spent

Quote (*non-SAPV, Participant 2*): “And then there's the time factor, so time is a very precious commodity in SAPV or in outpatient medicine in general, and it wouldn't be possible in practice to carry out ABS measures on patients. For me, the cost and benefit are out of proportion.”

- 2) Lack of staff

Quote (*SAPV, Participant 4*): “I would think it would be good, but I don't think there's anyone who can do it or anyone you can get.”

- 3) Lack of diagnostic options

Quote (*SAPV, Participant 13*): “To be honest, I don't think that has any value in SAPV. We can't even carry out the diagnostics that are crucial for ABS.”

- 4) Low medical significance relative to SAPV

Quote (*SAPV, Participant 5*): “The disadvantage is that there is too much bureaucracy for my taste. I want to keep palliative care as straightforward and narrow as possible. There is more of a risk of over-therapizing and saying that we wouldn't have done any antibiotics at all and now they are coming with the special antibiotic, and I would be worried that this could lead to over-therapy. I don't see the need at all.”

- 5) Lack of necessity

Quote (*SAPV, Participant 4*): “So we have to be careful not to stylize a problem area without commenting on it too negatively, but simply to say that it is not such a central component.”

- 6) Benefits for only a few patients within SAPV

According to two interviewees, only 3%–5% of patients would benefit from an ABS system.

Quote (*SAPV, Participant 5*): “…And I don't think it really makes sense because of the frequency. … and I think that the objective here is different in the palliative area. Given the small number of antibiotics administered, there's no need for yet another instrument to stir things up. For me, there would be no relation between benefit and effort.”

One participant was also critical of the fact that “there are many palliative care physicians who are very critical of it and tend to say, no, it's no good, it only prolongs life and it doesn't make any sense” (Quote *SAPV, Participant 14*)*.* This “certainly seems to be one of the biggest hurdles to encountering a group that rejects these antibiotics from the outset” (Quote *SAPV, Participant 8*)*.*

The “constant further training” that would be required for ABS measures is also perceived as more of a burden, because “it's not something you do once and stop, it's constant, you have to stay on the ball” (Quote *SAPV, Participant 1*).

The interviewees considered further education and training measures to be particularly important ABS measures for use in SAPV, so that “people are always aware of the current status, so to speak, that this happens regularly” (Quote *non-SAPV, Participant 2*)*.* A fixed contact person was also considered important. In contrast, patient education and screening about antibiotics were only occasionally rated as an important measure.

### Evaluation of potential benefits of ABS measures in SAPV

**Fig. 1:**
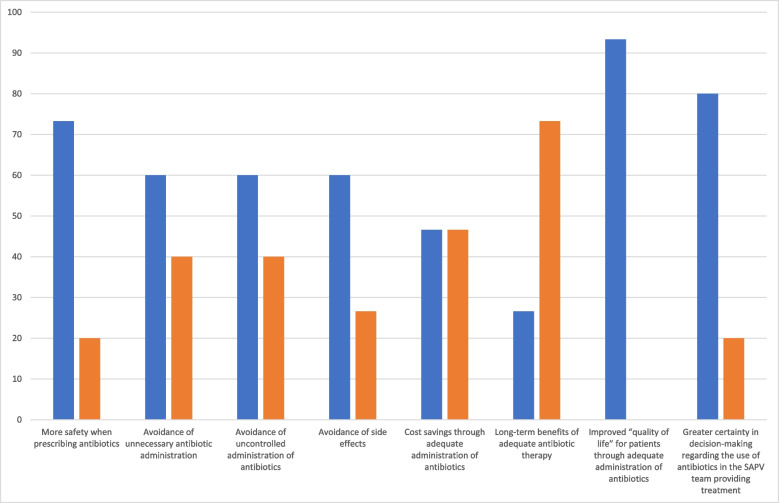
Responses regarding the applicability/non applicability of the benefits of an ABS system/measures in SAPV. Responses in percent; applicability in blue; non applicability in orange

The interviewees were asked to rate the possible advantages of the ABS system/measures in SAPV as “Applicable” or “Not applicable” (Table 2; Fig. 1). Abstentions were possible.

**Table 2:** Assessment of whether the benefits of ABS system/measures in SAPV Apply or do Not Apply

Advantages of ABS system/measures		
*Possible advantage*	*Applicable*	*Not applicable*
More safety when prescribing antibiotics	11	3
Avoidance of unnecessary antibiotic administration	9	6
Avoidance of uncontrolled administration of antibiotics	9	6
Avoidance of side effects	9	4
Cost savings through adequate administration of antibiotics	7	7
Long-term benefits of adequate antibiotic therapy	4	11
Improved “quality of life” for patients through adequate administration of antibiotics	14	0
Greater certainty in decision-making regarding the use of antibiotics in the SAPV team providing treatment	12	3

The overview of the applicable benefits primarily showed improved quality of life for patients and increased safety for the use and prescription of antibiotics by the SAPV team.

The overview of the “Not applicable” benefits revealed little agreement for the long-term benefits of adequate antibiotic therapy and the cost savings from the adequate administration of antibiotics in particular.

### Potential quality improvement

Eleven of the interview participants observed an improvement in the quality of patient care through the introduction of ABS measures in SAPV, whereas four participants did not. However, the introduction of such innovations “must be done with a sense of proportion” (quote *non-SAPV, Participant 7*), because “if the conditions are there, then quality can improve” (quote *SAPV, Participant 8*), because “you simply get more certainty and can already avoid grossly wrong decisions” (quote *SAPV, Participant 1*).

### Organizational aspects of implementing ABS in SAPV

According to the interviewees, ABS measures may be implemented into SAPV, particularly by training the SAPV team. This can take place, for example, “via external supervision” (quote *non-SAPV, Participant 10*), via an “extra team meeting or in coordination with the physicians” (quote *SAPV, Participant 8*), through “a training group” (quote *non-SAPV, Participant 10*), or “an external contact person if you have any queries” (quote *SAPV, Participant 13*). Designating a specific trained person in the SAPV team was also considered a good option for implementation.

With regard to the initiation and organization of ABS measures in SAPV, the interviewees believed that “the managing director must first decide whether it should be done or not” (quote *SAPV, Participant 4*). ABS should be “initiated/organized by a nurse as well as a physician” (quote *SAPV, Participant 11*)*.* The other individuals responsible for the organization would be the SAPV physician in charge, the SAPV coordinator, and the hygiene officer.

The question of whether ABS measures should also take the form of regular (online) team conferences received strong support from all 15 respondents. The reasons provided for an online format included: 1) the usual team meetings already taking place online and including the ABS topic would be possible with little effort, 2) online meetings primarily save time, and 3) could be done independent of location. The frequency of such online meetings recommended by the participants was once a month (mentioned most frequently), once a quarter, or once every six months.

According to 14 of the 15 interviewees, only problem cases with bacterial infections should be discussed using ABS measures. Only one respondent recommended including a discussion for all patients with bacterial infections.

## Discussion

International guidelines recommend the differential use of antibiotics for the treatment of palliative patients [15]. In contrast to palliative inpatient care, in which ABS teams are already firmly integrated in some cases to support the appropriate use of antibiotics, the outpatient sector is a largely unexplored area. This is reflected in the existing data situation. Although 76 publications on the topic of “Antibiotic Stewardship palliative care” appeared in the PubMed database in the period 2014–2024, there are only three publications on “ABS specialized outpatient palliative care,” of which only one focused on the outpatient sector. In Germany, approximately 10% of palliative care patients are treated by SAPV [3]. As this patient group experiences complex symptoms, we examined the extent to which this patient group may benefit from the introduction of ABS measures to SAPV. There were no significant differences between the SAPV and non-SAPV member groups with respect to their opinions during the interview.

Early integration of palliative care for the treatment of seriously ill patients has not only benefited those affected [22, 23], but also broadened the concept of palliative care. Palliative care encompasses a more or less long period of life and can begin as a merely supportive service and become the primary mode of care at the end of life [24–26].

For SAPV, patients can also be assigned to different palliative phases (stable, deteriorating, unstable, deceased) analogous to the PCOC Symptom Assessment Scale [27], which can overlap in some cases [28]. From this perspective, the use of antibiotics in the context of palliative care must be considered on an individualized basis [24]. While, in the opinion of the experts interviewed – as well as reports in the literature [29] – in the early days of palliative care antibiotic administration can be a life-prolonging measure in addition to symptom relief and thus an improvement in quality of life, the latter becomes increasingly less important as the disease progresses [30]. In contrast to the early stage of palliative care, when the goal of antibiotic therapy is to cure an infection, the alleviation of (infection)symptoms becomes decisive in the further course of the disease [16]. Consistent with the opinion of the interviewees, antibiotics are of little value during the final phase as they could do more harm to the patient because of potential side effects [16, 24, 31]. Nevertheless, antibiotic therapy is often continued until the patient dies [16].

In addition, the patient’s wishes, the opinions of their relatives, and especially in the outpatient sector, co-treating physicians, play an important role. Therefore, it may be difficult in certain situations to make a sensible decision for the patient regarding the use of antibiotics; thus, supportive options such as ABS measures may be helpful [16]. In the area of SAPV care, there is – as the experts point out – uncertainty in specific complex treatment situations with respect to the use/discontinuation of antibiotics, taking into account the groups of people involved. The majority of interviewees agree with this potential gain in safety in decision-making with regard to the use of antibiotics by introducing an ABS system into SAPV. While cost savings through the targeted/avoidable administration of antibiotics are considered significantly important in the literature [32], only approximately half of the experts surveyed considered it an advantage with regard to the introduction of an ABS measure in SAPV.

In contrast to inpatient treatment, antibiotics can usually only be administered at home orally, or in a few cases, intravenously or subcutaneously [33, 34]. In addition, diagnostic procedures, such as imaging, blood cultures, and laboratory tests are lacking or are only possible at great expense to the patient and the team providing care. As a result, antibiotic therapy is usually carried out without pathogen detection, which makes the indication and choice of antibiotics more difficult. With regard to “not prolonging life,” the use of antibiotics should always take into account the patient’s wishes and condition and, if possible, the choice should be agreed upon by relatives to avoid later disputes and accusations. The discussion point “Greater certainty in decision-making regarding the use of antibiotics in the SAPV team providing treatment,” of which 12 interview participants agreed with, should also be understood from this perspective.

Consistent with the inpatient sector [8], the interview participants indicated that an ABS team in SAPV would have the task of critically reviewing the use and duration of antibiotic therapy to avoid unnecessary use. According to some interviewees, however, only a few patients would benefit from an ABS measure in an SAPV program. A similar approach has been discussed for the use of antiproliferative agents in inpatient hospice, although a selected small group of palliative care patients benefited from the expertise of a corresponding expert [35]. Something similar would be conceivable when using an ABS expert/ABS measures in SAPV for selected complex cases. A special group of patients in the field of palliative care in particular can benefit from the expertise of an ABS expert: patients with infections that can no longer be treated curatively and who require long-term antibiotic therapy (so-called suppression therapy, such as for infected intravascular devices or vascular prostheses). Various concepts have been applied in which antibiotics can be used intravenously and orally. The selection depends on the possibilities of on-site administration and the causative pathogen [36]. Newer antibiotics in particular can be used in these situations [37].

The interviewees considered ABS measures in the area of further training or the addition of an ABS expert to be particularly useful, as this could improve the quality of patient care similar to the inpatient area [16]. However, the permanent implementation of ABS measures in SAPV was considered difficult by the experts for reasons such as staff shortages, insufficient deployment options, and a lack of time. This is in marked contrast to inpatient care, where the introduction of ABS measures is already more advanced, despite the shortage of skilled staff [38]. This problem was also highlighted by the German Medical Association’s Drug Commission back in 2017 [39] and still exists today, which makes it difficult to implement new structures.

## Conclusion

Overall and according to the experts interviewed, the implementation of ABS measures in SAPV, similar to what has already been done in nursing homes [14], is considered feasible in principle, but difficult to implement under the current conditions. Close cooperation with an existing external ABS expert/team could prove helpful. This would provide more security for a small, but still relevant proportion of SAPV patients and for the SAPV treatment team. Focusing on complex patients with bacterial infections may also be a useful first step toward implementing such measures. The increased development of web-based communication structures since the COVID-19 pandemic is considered a positive factor for such implementation. The practical implementation and measurement of the potential benefits of introducing ABS structures into SAPV should be analyzed in further studies.

## Limitations

This study had some limitations. For example, only one SAPV was surveyed, which involved care for patients in rural areas. Thus, issues with care may differ from urban areas. These points should be considered in future studies.

## Supplementary Information

Supplementary Material 1.

## Data Availability

All relevant data are within the paper. The transcripts analyzed in this study are available from the corresponding author on reasonable request.

## Abbreviations

ABS: Antibiotic stewardship
IMIG: Institute for Market Research in Healthcare Munich
SAPV: Specialized outpatient palliative care
