# Supplementary material for: Is the use of antibiotic stewardship measures in the context of specialized outpatient palliative care sensible and feasible? An interview-based study
Source: BMC Palliat Care. 2024 Dec 7;23:280. doi: 10.1186/s12904-024-01609-x (PMC11624597; doi:10.1186/s12904-024-01609-x)
Supplement: Supplementary file 1 — Supplementary Material 1. [file 12904_2024_1609_MOESM1_ESM.pdf]

Interview guide used

**Entry questions**	
Question 1	How do you personally feel about the use of antibiotics in palliative care (early phase vs. final phase)?
Question 2	In your opinion, are there differences in the use of antibiotics in a palliative or curative setting?
Question 3	What are the most important aspects for you when using antibiotic therapy in palliative care?
Question 4	What advantages and/or disadvantages do you see in the use of antibiotics in palliative care?
**Use of antibiotics in SAPV**	
Question 5	How do you generally view the use of antibiotics in SAPV in particular?
Question 6	Is there a difference between patients in SAPV and nonpalliative outpatient care?
Question 7	What advantages and/or disadvantages do you see in the use of antibiotics in SAPV?
Question 8	Which benefit/risk assessment should be decisive for the use of antibiotics in SAPV?
Question 9	When the patient is admitted to SAPV, should the existing antibiotic therapy be maintained/continued or adapted/changed by SAPV?
Question 10	If adapted: What should the adjustment be based on?
Question 11	If there are differing views on the use of antibiotics between the SAPV team on the one hand and the patient, the patient's relatives, or the patient's family doctors/specialists providing parallel treatment on the other, how should this be dealt with? What would be possible solutions?

Question 12	Would you generally still admit a patient from SAPV as an inpatient due to an infection?
**Attitude and evaluation of Antibiotic Stewardship (ABS)**	
Question 13	What do you think about ABS based on the description you have just read?
Question 14	Have you personally had any experience with ABS measures and if so, what kind?
Question 15	What key benefits do you see for participants in the ABS training courses and the ABS measures taught?
Question 16	What central advantages or possible disadvantages do you see for the patient in the ABS measures applied?
**Pros and cons of ABS system/ABS measures in SAPV**	
Question 17	What do you generally think about the implementation of an ABS system/ABS measures in SAPV?
Question 18	What obstacles/barriers do you see in the regular implementation of ABS measures in SAPV?
Question 19	What do you consider to be particularly important ABS measures for use in SAPV?
Question 20	How could ABS measures best be implemented/introduced in SAPV?
Question 21	Who should initiate and organize the ABS measures/ABS system in SAPV?
Question 22	Should the ABS measures also take the form of regular (online) team conferences? If yes: What are the reasons for this? How often? If no: What speaks against it?
Question 23	Should all SAPV patients with bacterial infections be discussed using the ABS measures or only “random sample patients” or only the problem cases?

Question 24	How do you see/rate (applicable or rather not applicable) the following advantages of an ABS system (ABS measures) in SAPV**:**<br>- More safety when prescribing antibiotics<br>- Avoidance of unnecessary antibiotic administration<br>- Avoidance of uncontrolled administration of antibiotics<br>- Avoidance of side effects<br>- Cost savings through the adequate administration of antibiotics<br>- Long-term benefits of adequate antibiotic therapy<br>- Improved “quality of life” for the patient due to adequate administration of antibiotics<br>- Greater certainty in decision-making regarding the use of antibiotics in the treating SAPV team
Question 25	What other advantages and/or disadvantages do you see with an ABS system (ABS measures) in SAPV?
**Closing question**	
Question 26	In your opinion, can the implementation of an ABS system (ABS measures) in SAPV improve the quality of patient care?
